# The Effect of Smoking on Endothelial Dysfunction in Autosomal Dominant Polycystic Kidney Disease Patients with Preserved Renal Function

**DOI:** 10.1080/0886022X.2021.1949348

**Published:** 2021-07-13

**Authors:** Cuma Bulent Gul, Abdulmecit Yildiz, Saim Sag, Aysegul Oruc, Alparslan Ersoy, Sumeyye Gullulu

**Affiliations:** a Department of Nephrology, Bursa Yuksek Ihtisas Training and Research Hospital, Bursa, Turkey; b Department of Nephrology, Uludag University Faculty of Medicine, Bursa, Turkey; c Department of Cardiology, Acibadem University Faculty of Medicine, Istanbul, Turkey; d Department of Cardiology, Uludag University Faculty of Medicine, Bursa, Turkey

**Keywords:** Autosomal dominant polycystic kidney disease (ADPKD), smoking, normal kidney function, endothelial dysfunction

## Abstract

**Background:**

In autosomal dominant polycystic kidney disease (ADPKD), endothelial dysfunction (ED) is common and occurs much earlier than kidney function impairment. The impact of smoking on ED in ADPKD patients has not been previously studied. The aim of this study was to investigate the potential contribution of smoking habits to ED and subclinical atherosclerosis in these patients.

**Methods:**

This case-control study included 54 ADPKD patients with preserved renal function and 45 healthy control subjects. ED was assessed using ischemia-induced forearm flow-mediated dilatation (FMD). Carotid intima-media thickness (CIMT) was measured from 10 mm proximal to the right common carotid artery. Clinical demographic characteristics and laboratory data were recorded for the patients and control group. Regression analysis was used to determine independent associations of ED and CIMT.

**Results:**

FMD was significantly lower in the ADPKD patients (19.5 ± 5.63 vs. 16.56 ± 6.41, *p* = .018). Compared with nonsmoker ADPKD patients, smoker patients had significantly lower FMD values (18.19 ± 6.52 vs. 13.79 ± 5.27, *p* = .013). In multiple regression analysis, age (*β* = –0.294, 95% CI: −0.392: −1.96, *p* = .001) for FMD and smoking (*β*  =  1.328, 95% CI: 0.251, 2.404, *p* = .017) for CIMT were independent predictors.

**Conclusions:**

Patients with ADPKD had more impaired endothelial function and subclinical atherosclerosis compared with control subjects. Smoking may increase the risk of subclinical atherosclerosis in ADPKD patients.

## Introduction

Autosomal dominant polycystic kidney disease (ADPKD) is the most prevalent heritable kidney disease. This condition may progress as far as the phase of end-stage renal disease in some patients. Moreover, ADPKD patients also have increased cardiovascular morbidity compared with their non-ADPKD counterparts who have functional kidney impairment of similar magnitude [1]. Endothelial dysfunction (ED) is considered to be the earliest discernable phase of the atherosclerotic process [2,3]. ED is seen very early during the course of ADPKD, long before renal function starts to deteriorate. Although many factors ranging from disease-specific abnormalities to nontraditional risk factors have been held responsible for the development of ED in the early phase of ADPKD, the main cause of ED is still unclear [4].

Traditional cardiovascular risk factors are prevalent in patients with ADPKD and negatively affect disease progression. Smoking is one of these factors, which has been associated with CKD progression in ADPKD patients [5]. Smoking is a preventable risk factor contributing to CVD in the renal and non-renal population but has not been specifically investigated in ADPKD patients [6]. Smoking leads to ED through the production of significant quantities of free oxygen radicals and pro-oxidant molecules [7]. To the best of our knowledge, there is no study in the literature that has assessed the effects of smoking on endothelial function and subclinical atherosclerosis through evaluations of carotid intima-media thickness (CIMT) in patients with ADPKD. Therefore, the aim of this study was to evaluate the impact of active cigarette smoking on endothelial function and CIMT in prevalent ADPKD patients in a case-control design.

## Subjects and methods

This case-control study included patients from the Nephrology Outpatient Clinic of a university teaching hospital. The study included patients with known ADPKD and healthy control subjects. ADPKD diagnosis was determined based on ultrasound and family history, as described by Pei et al. [8]. The patients included were only those with relatively preserved kidney function with estimated glomerular filtration rate (eGFR) values >60 mL/min/1.73 m^2^. The highest value of serum creatinine was 1.3 mg/dL in the ADPKD group. ADPKD patients with diabetes mellitus and/or known previous cardiovascular disease (previously documented myocardial infarction, congestive heart failure, stroke, peripheral vascular disease) were excluded from the study. The control subjects were selected from patients who had been evaluated in the Nephrology Outpatient Clinics with suspicion of hypertension (HT), but ambulatory blood pressure monitoring records excluded HT. None of the control subjects had diabetes mellitus or any other documented CV disease. Routine biochemistry studies, as well as standard blood counts, were performed using auto analyzers in the central hospital laboratory. An experienced cardiologist performed the carotid intima-media and flow-mediated dilatation (FMD) measurements as described in more detail below for both the patient and control groups. eGFR was calculated based on serum creatinine values with the CKD-EPI equation [9]. Demographic characteristics, medication use, and smoking history were recorded for both groups and blood pressure was recorded. CIMT was measured from 10 mm proximal to the right common carotid artery bifurcation segment, using the same ultrasound device, which was used to measure FMD with a 12 MHz linear-array imaging probe.

### Flow-mediated dilatation

In all the patients and control subjects, arterial blood pressure was determined in the morning by a cardiologist taking three consecutive measurements. Each measurement was performed after a 15-min rest period and the mean values were calculated for systolic and diastolic pressures. Endothelium-dependent FMD was assessed non-invasively in the brachial artery using high-resolution ultrasound [10]. The measurements were performed by a single observer with a widely available transducer and equipment (12 MHz linear-array imaging probe, Vivid 7, GE-Vingmed, Horten, Norway). The participants remained in the supine position for at least 15 min before the examination was started. The subject’s arm was comfortably immobilized in the extended position to allow consistent recording of the brachial artery at 2–4 cm above the antecubital fossa. Three adjacent measurements of end-diastolic brachial artery diameter were taken from single two-dimensional frames. A pneumatic tourniquet was inflated up to 200 mmHg to obliterate the radial pulse. After 5 min, the cuff was deflated. Flow measurements were performed 60 s after the deflation. The FMD was calculated as the percentage change in diameter compared with baseline resting diameters.

### Statistical analysis

Data obtained in the study were analyzed statistically using IBM SPSS Statistics v.20 software (Armonk, NY). Conformity of the data to normal distribution was assessed using the Shapiro–Wilk test. Descriptive statistics were reported as mean ± standard deviation values for normally distributed variables and median ± interquartile range values for non-normally distributed variables. Categorical variables were reported as frequency and percentage. The independent samples *t*-test, one-way ANOVA, Mann–Whitney’s *U* test, and the Kruskal–Wallis test were used for intergroup comparisons. Bonferroni’s *post hoc* test was used to evaluate statistical significance after ANOVA. The Pearson Chi-square and Fisher’s exact tests were also applied in intergroup comparisons. Multiple linear regression analysis, using the backward elimination method, was performed in order to explore independent relationships between FMD (model A) and CIMT (model B) in the ADPKD group, and in the whole cohort. Age, BMI, smoking duration, eGFR, serum creatinine, hsCRP, cholesterol, HDL-C, LDL-C, triglyceride, uric acid, and ambulatory systolic and diastolic averages were placed in the regression analysis model as independent variables since they play a significant role in endothelial function. A value of *p* < .05 was considered statistically significant. Post hoc power analysis for CIMT and FMD was performed using a web-based ‘E-PICOS’ program. For the alpha error value of 0.05, means and standard deviations of the group and the sample size of each group were entered into the program, and calculations were made.

The study protocol was approved by the Ethics Committee of Uludag University Medical Faculty (document no.: 12/94-09). Written informed consent was obtained from all the study subjects.

## Results

Evaluation was made of 54 ADPKD patients and 45 control subjects. In the ADPKD patient group, 20 were active smokers at the time of inclusion in the study. The clinical and demographic characteristics and laboratory data of the patients are shown in Tables 1 and 2. The prevalence of smoking was similar in the ADPKD patients and control subjects. No difference was determined between the groups in respect of body mass index (BMI) and gender distribution. The ADPKD patients and healthy nonsmokers had significantly higher age (Table 1). The median eGFR values of the ADPKD smokers were significantly lower than those of the nonsmoker ADPKD patients and the control subjects (Table 2).

**Table 1.** Clinicodemographic characteristics of patients.

Parameter	ADPKD Smoker (*n* = 20) (I)	ADPKD Nonsmoker (*n* = 34) (II)	Healthy Smoker (*n* = 19) (III)	Healthy Nonsmoker (*n* = 26) (IV)
Age	45.5 (40–55)^a,b^	38.5 (28–55)	39.0 (37–60)^b^	39.0 (30–48)
Gender (F/M)	9/11	20/14	7/12	12/14
Current smokers	20 (37%)	NA	19 (42%)	NA
Smoking duration (years)	12 (8–25)	NA	11 (7–24)	NA
BMI (kg/m^2^)	27.4 (24.0–30.0)	25.5 (22.5–28.2)	22.3 (20.8–27.0)	21.0 (20.0–23.0)
Hypertension (*n*%)	8 (40.0%)	18 (52.9%)	NA	NA
Drug use	9 (45.0%)	14 (42.4%)	NA	NA
ACE inhibitor	4 (20.0%)	5 (14.7%)	NA	NA
ARB	4 (20.0%)	9 (26.5%)	NA	NA
CCB	7 (35.0%)^a^	3 (8.8%)	NA	NA
Beta-blocker	5 (25.0%)	4 (11.8%)	NA	NA
Diuretic	3 (15.0%)	4 (11.8%)	NA	NA

BMI: body mass index; ACE: angiotensin-converting enzyme; ARB: angiotensin receptor blocker; CCB: calcium channel blocker.

^a^ *p* < .05 vs. group II.

^b^ *p* < .05 vs. group IV.

**Table 2.** Various laboratory parameters and endothelial measurements.

Parameter	ADPKD Smoker (*n* = 20) (I)	ADPKD Nonsmoker (*n* = 34) (II)	Healthy Smoker (*n* = 19) (III)	Healthy Nonsmoker (*n* = 26) (IV)
Microalbumin (µg/min)	41 (13–79)	19 (10–45)	NA	NA
eGFR (mL/min)	97.55 (76–107)^b,c^	107.10 (81–120)	109.47 (95–121)	109.50 (96–125)
hs-CRP (mg/L)	0.51 (0.36–0.88)^b,c^	0.36 (0.33–0.67)^b,c^	0.30 (0.26–0.41)	0.34 (0.23–0.40)
Fasting blood glucose (mg/dL)	89.5 (84–104)	87.0 (81–91)	81.0 (77–87)	81.5 (76–88)
Creatinine (mg/dL)	0.98 (0.71–1.16)^a,b,c^	0.77 (0.69–0.96)	0.70 (0.64–0.85)	0.77 (0.72–0.87)
Uric acid (mg/dL)	5.75 (4.5–7.6)^b,c^	4.85 (4.3–6.7)^b,c^	2.90 (2.5–3.3)	3.00 (2.6–3.6)
Albumin (g/dL)	4.21 ± 0.28	4.27 ± 0.29	4.28 ± 0.26	4.25 ± 0.30
Cholesterol (mg/dL)	205.80 ± 38.67	189.50 ± 40.42	177.31 ± 23.75	175.65 ± 25.39
HDL cholesterol (mg/dL)	43.15 ± 10.91	44.82 ± 9.40	43.00 ± 6.50	47.50 ± 10.26
Triglyceride (mg/dL)	132 (91–165)	119 (63–168)	113 (78–158)	115 (87–150)
LDL cholesterol (mg/dL)	122 (93–161)	119 (101–134)	107 (93–138)	94 (74–119)
Hemoglobin (g/dL)	14.1 (13.0–15.8)	13.2 (12.7–14.7)	13.6 (12.6–14.6)	12.6 (11.7–13.6)
RDW	15.10 ± 1.33	14.59 ± 1.25	14.58 ± 1.55	16.13 ± 2.04
NLR	2.44 ± 1.36	2.31 ± 0.81	2.29 ± 0.77	2.20 ± 0.90
Systolic BP (mmHg)	115.30 ± 7.47^b,c^	115.21 ± 10.54	113.42 ± 7.35	114.76 ± 7.49
Diastolic BP (mmHg)	69.95 ± 7.10^b,c^	67.77 ± 8.69^b^	64.63 ± 8.22	69.53 ± 5.51
CIMT (mm)	8 (5.5–10)^a,b,c^	6 (5–7.5)	6 (5–6.5)	5 (4–6)
FMD (%)	13.79 ± 5.27^a,b,c^	18.19 ± 6.52	20.32 ± 6.11	18.39 ± 4.83

hsCRP: high-sensitive C-reactive protein; eGFR: estimated glomerular filtration rate; HDL: high-density lipoprotein; LDL: low-density lipoprotein; RDW: red cell distribution width; NLR: neutrophil lymphocyte ratio; BP: blood pressure; CIMT: carotid intima-media thickness; FMD: flow-mediated dilatation.

Data are presented as mean ± SD for normally distributed parameters and as median+(interquartile range) for non-normal distributed parameters. Statistical significance corresponds to: ^a^*p* < .01 vs. group II, ^b^*p* < .01 vs. group III, and ^c^*p* < .01 vs. group IV.

The FMD values were significantly lower in ADPKD patients. A statistically significant correlation was determined between CIMT and smoking status in the ADPKD patients (*r* = 0.363, *p* = .005) but not in the control group (*r* = 0.246, *p* = .246). The CIMT values were higher in the smoker group (Table 2).

In the ADPKD group, 20 (37%) patients were active smokers. The frequency of HT was similar in the smoker and nonsmoker groups. Compared with nonsmoker ADPKD patients, smokers had significantly lower FMD and higher CIMT values. The patient and control groups were subdivided as smokers and nonsmokers to assess how smoking affects CV factors in smoker subgroups. The FMD and CIMT values of the smoker subgroup of the healthy control group were close to those of the nonsmoker subgroup of ADPKD patients (Table 2).

In multivariate regression analysis, the parameters of age, BMI, smoking duration, eGFR, serum creatinine, hsCRP, cholesterol, HDL-C, LDL-C, uric acid, and ambulatory systolic and diastolic averages were inserted as independent variables. In ADPKD patients, after backward elimination, age and smoking status were left in model A, but only age was found to be significant. In model B, age, smoking, BMI, HDL, and HT were left, and only smoking and HT were found to be significant for the prediction of CIMT. In the whole cohort, after backward elimination, only hsCRP was left in model A and model B, and this was found to be statistically significant in both models (Table 3).

**Table 3.** Multivariable linear regression analysis shows independent associations of flow mediated dilatation (FMD) and CIMT in the ADPKD group and whole cohort.

ADPKD (*n* = 54)			Whole cohort (*n* = 99)		
Model A (dependent variable FMD)					
(*R*^2^: 0.606, *p* < .005)			(*R*^2^: 0.614, *p* < .001)		
Independent variable	*β*	*p* Value	Independent variable	*β*	*p* Value
Age	–0.294	**.001**	hsCRP	–7.445	**.043**
Smoking	–2.340	.089	Smoking	–0.032	.058
			Microalbuminuria	–0.021	.053
Model B (dependent variable CIMT)					
(*R*^2^: 0.651, *p* < .001)			(*R*^2^: 0.482, *p* < .033)		
Independent variable	*β*	*p* Value	Independent variable	*β*	*p* Value
Age	0.40	.081	hsCRP	5.915	**.007**
Smoking	1.328	**.017**			
BMI	0.113	.093			
HDL	0.045	.057			
HT	1.036	**.046**			

FMD: flow-mediated dilatation; CIMT: carotid intima-media thickness; BMI: body mass index; HDL: high-density lipoprotein; HT: hypertension. Bold values are statistically significant at *p* < .05.

The backward elimination method was used in the linear regression model.

## Discussion

The most important finding of this study was that patients with ADPKD who had relatively preserved renal function had greater ED than healthy control subjects. Smoking was associated with more depressed endothelial function in ADPKD patients. The nonsmoker ADPKD patients had similar FMD values as the control subjects who were current smokers. However, only age remained as an independent predictor of FMD in multivariable regression analysis.

Cardiovascular disease is the main cause of death in patients with kidney disease [11]. This is also true for patients with ADPKD. However, ADPKD confers some additional risks apart from impaired kidney function. HT is prevalent in this patient population and precedes the development of CKD in 60% of patients [12]. Several factors come into play in the early development of HT in ADPKD, including activation of the renin–angiotensin–aldosterone system, sympathetic overactivity, increased plasma vasopressin and endothelin-1 among others [13,14]. Left ventricular hypertrophy is also prevalent and can be encountered even in patients whose blood pressures are within the upper quartile of the normal range [15].

Increased cardiovascular morbidity and mortality are undoubtedly also related to generalized ED and subsequent atherosclerotic disease processes such as CIMT in ADPKD. One of the central pathophysiological disorders, namely dysregulation of the primary cilium, may lead to endothelial and vascular smooth muscle cell dysfunction [4]. Interestingly, changes in the vascular endothelium and smooth muscle layer occur prior to the clinical findings of ADPKD. In a small study by Borresen et al. [16], pulse wave reflection was shown to be amplified in normotensive patients with ADPKD and preserved renal function. Endothelium-dependent relaxation has been reported to be impaired together with reduced endothelial NO synthase activity in ADPKD [17]. However, the contribution of classical CV risk factors such as HT, dyslipidemia, and smoking cannot be ignored.

Although the reasons for early-impaired ED have not been fully elucidated as yet, several studies have pointed to the role of increased oxidative stress [18,19].

Smoking is an established traditional cardiovascular risk factor and is related to increased mortality globally [20]. Smoking may enhance adverse consequences in several ways in ADPKD patients. Smoking is associated with increased LDL cholesterol levels and has a negative effect on vascular stiffness [21,22]. It has also been reported that smoking leads to ED mainly due to the production of large amounts of free radicals and pro-oxidant molecules [7].

The effects of stopping smoking also support the impact of smoking on endothelial function. A prospective, double-blind, randomized, placebo-controlled clinical trial of 1504 current smokers showed that 36.2% of participants who stopped smoking had an improvement in FMD measurements, whereas there was no change in FMD values of those who continued smoking [23]. Smoking also has detrimental effects on kidney function [23,24]. However, to the best of our knowledge, no study to date has attempted to investigate the effects of smoking on endothelial function in patients with ADPKD. Smoking has been shown to increase the acute phase response by stimulating the systemic inflammatory response [25].

The findings of the current study showed that in line with previous studies, hsCRP was significantly higher in ADPKD patients compared with healthy control subjects. Although, hsCRP predicted FMD and CIMT in the whole group, it had no predictive value in ADPKD patients. This result could be due to the low number of patients in the ADPKD group. Increased CRP levels have been found to be associated with ED in normotensive ADPKD patients [25].

The current study results showed for the first time that smoking might have deleterious effects on endothelial function in ADPKD patients. Smoking was associated with lower FMD values in ADPKD patients who were current smokers compared to nonsmokers. LDL cholesterol was also higher in these patients. Therefore, smoking may deteriorate the already impaired endothelial function and increase global cardiovascular risk in ADPKD patients.

There were some limitations to this study. First, post hoc power analysis revealed that the power of the study was 66% for FMD and 99% for CIMT. Although the power was sufficient for CIMT, it was moderately low for FMD, compared to the level of 80% recommended by Cohen [26]. Second, quantitative data (number of pack-years) of cigarette smoking for the control subjects and some ADPKD patients were not available. Another limitation was that oxidative system markers were not evaluated so no comment can be made regarding the possible underlying mechanism of the association between smoking and FMD. Finally, smokers in both the ADPKD group and control group were significantly older compared with their respective nonsmoker counterparts. Moreover, age remained as the single significant determinant of FMD in multivariable analysis. Similarly, smokers had lower eGFR values compared with nonsmokers, although eGFR was not an independent determinant of FMD. The current study results showed smoking and HT to be the main determinants of CIMT. Although these findings would not be surprising in the normal population, they demonstrate the importance of modifiable CV risk factors in the pathogenesis of subclinical atherosclerosis. In ADPKD patients, in addition to the constant genetic factors, environmental factors such as smoking elimination may positively affect prognosis

## Conclusions

This is the first study in the literature to have examined the effect of smoking on endothelial function in ADPKD patients. ADPKD patients who were active smokers were seen to have significantly higher CIMT values, which are signs of early atherosclerosis, compared with their nonsmoker counterparts. Nevertheless, there is a need for further studies with larger sample size and possibly with measurements of some oxidative system markers to be able to shed further light on this issue.

## Ethical approval
